# Serological Evidence of Cryptic Rift Valley Fever Virus Transmission Among Humans and Livestock in Central Highlands of Kenya

**DOI:** 10.3390/v16121927

**Published:** 2024-12-17

**Authors:** Silvia Situma, Evans Omondi, Luke Nyakarahuka, Raymond Odinoh, Marshal Mweu, Marianne W. Mureithi, Martin M. Mulinge, Erin Clancey, Jeanette Dawa, Isaac Ngere, Eric Osoro, Bronwyn Gunn, Limbaso Konongoi, Samoel A. Khamadi, Johan Michiels, Kevin K. Ariën, Barnabas Bakamutumaho, Robert F. Breiman, Kariuki Njenga

**Affiliations:** 1 Department of Medical Microbiology and Immunology, University of Nairobi, Nairobi 00202, Kenya; marianne@uonbi.ac.ke; 2 Washington State University Global Health Program-Kenya, Nairobi 00200, Kenya; raymond.odinoh@wsu.edu (R.O.); jdawa@cartafrica.org (J.D.); isaac.ngere@wsu.edu (I.N.); eric.osoro@wsu.edu (E.O.); 3 Department of Animal Science, Pwani University, Kilifi 80108, Kenya; 4 African Population and Health Research Center (APHRC), Nairobi 00100, Kenya; evansotieno@aims.ac.za; 5 Institute of Mathematical Sciences, Strathmore University, Nairobi 00200, Kenya; 6 Uganda Virus Research Institute, Entebbe P.O. Box 49, Uganda; nyakarahuka@gmail.com (L.N.); bbarnabas2001@yahoo.com (B.B.); 7 Department of Animal Resources and Biosecurity, Ecosystems and Veterinary Public Health, Makerere University, Kampala P.O. Box 7062, Uganda; 8 Department of Public Health, University of Nairobi, Nairobi 00202, Kenya; marshal@uonbi.ac.ke; 9 Department of Biochemistry, University of Nairobi, Nairobi 00100, Kenya; mmulinge@uonbi.ac.ke; 10 Paul G. Allen School for Global Health, Washington State University, Pullman, WA 98165, USA; erin.clancey@wsu.edu (E.C.); bronwyn.gunn@wsu.edu (B.G.); 11 Center for Epidemiological Modelling and Analysis, University of Nairobi, Nairobi 00202, Kenya; 12 Kenya Medical Research Institute, Nairobi 00200, Kenya; limbaso@gmail.com (L.K.); skhamadi@kemri.go.ke (S.A.K.); 13 Virology Unit, Department of Biomedical Sciences, Institute of Tropical Medicine, 2000 Antwerp, Belgium; jmichiels@itg.be (J.M.); karien@itg.be (K.K.A.); 14 Rollins School of Public Health, Emory University, Atlanta, GA 30322, USA; rfbreiman@emory.edu

**Keywords:** arbovirus, cryptic transmission, Rift Valley fever virus, non-epidemic period

## Abstract

Although the highlands of East Africa lack the geo-ecological landmarks of Rift Valley fever (RVF) disease hotspots to participate in cyclic RVF epidemics, they have recently reported growing numbers of small RVF clusters. Here, we investigated whether RVF cycling occurred among livestock and humans in the central highlands of Kenya during inter-epidemic periods. A 2-year prospective hospital-based study among febrile patients (March 2022–February 2024) in Murang’a County of Kenya was followed by a cross-sectional human–animal survey. A total of 1468 febrile patients were enrolled at two clinics and sera tested for RVF virus RNA and antiviral antibodies. In the cross-sectional study, humans (n = 282) and livestock (n = 706) from randomly selected households were tested and questionnaire data were used to investigate sociodemographic and environmental risk factors by multivariate logistic regression. No human (n = 1750) or livestock (n = 706) sera tested positive for RVFV RNA. However, 4.4% livestock and 2.0% humans tested positive for anti-RVFV IgG, including 0.27% febrile patients who showed four-fold IgG increase and 2.4% young livestock (<12 months old), indicating recent virus exposure. Among humans, the odds of RVF exposure increased significantly (*p* < 0.05, 95% CI) in males (aOR: 4.77, 2.08–12.4), those consuming raw milk (aOR: 5.24, 1.13–17.9), milkers (aOR: 2.69, 1.23–6.36), and participants residing near quarries (aOR: 2.4, 1.08–5.72). In livestock, sheep and goats were less likely to be seropositive (aOR: 0.27, 0.12–0.60) than cattle. The increase in RVF disease activities in the highlands represents a widening geographic dispersal of the virus, and a greater risk of more widespread RVF epidemics in the future.

## 1. Introduction

Rift Valley fever virus (RVFV), a zoonotic arbovirus belonging to the *Phenuiviridae* family, was first detected in Kenya in 1930 and is associated with acute febrile illness in humans and episodic abortions and juvenile mortality in livestock [1,2,3,4]. This mosquito-borne disease is a One Health global threat that has caused extensive public health and economic devastations in Africa and the Middle East [5,6,7,8,9]. The epidemiology of RVFV disease is marked by large epidemics followed by inter-epidemic periods (IEPs) during which clinical disease in livestock and humans are rarely reported [10]. While the disease has been well studied during epidemics documenting key geographic and ecological factors associated with disease occurrence, there is a limited understanding of virus maintenance including enabling environmental and climatic factors and the burden of disease among livestock and humans during IEPs [5,6,7,11,12,13,14,15]. These knowledge gaps hinder the establishment of effective surveillance, prevention strategies as well as adequate public health interventions and educational messaging.

The East Africa (EA) region has recently reported increasing frequency of small RVFV clusters in previously unaffected areas, associated with a combination of higher temperature and rainfall [16]. The western highlands in Kenya and Uganda seem to have a particularly unique profile favorable for these RVF clusters [17,18]. With elevations exceeding 1200 and 1900 m above sea level in Kenya and Uganda, respectively [5,16,17,19], these highlands are not among typical RVFV hotspots, which are characterized by low elevation (<500 m above sea level), arid and semi-arid conditions, and susceptibility to flooding [18].

Previous analysis of long-term climatic trends in the EA region revealed an annual mean temperature increase of 0.12 to 0.3 °C per decade in parts of East Africa, indicating a rise in annual average temperatures up to 1.2 °C over the past 40 years, contributing to escalating frequency and severity of droughts in the arid and semi-arid lowlands [16,20,21]. In the highlands of southwestern Uganda and Kenya, rising temperatures accompanied by increasing rainfall trends were associated with significantly increasing frequencies of RVF clusters [16]. This emerging evidence of increasing RVF disease activities in the highlands and other novel geographical settings suggests an increase in the spread of the virus and a greater risk of widespread RVF epidemics in the future. Here, we investigated a cryptic RVF virus maintenance cycle existing in the EA highlands associated with a sustained prevalence of RVFV in humans and livestock.

## 2. Materials and Methods

### 2.1. Study Design

A prospective two-year hospital-based study was conducted on febrile patients between March 2022 and February 2024, followed by a 10-day community-based human–animal linked cross-sectional study among randomly selected households in August 2023 in Murang’a County, central Kenya. The objective was to investigate the seroprevalence of RVFV and the factors associated with seropositivity in humans and livestock.

### 2.2. Study Area

**Figure 1:**
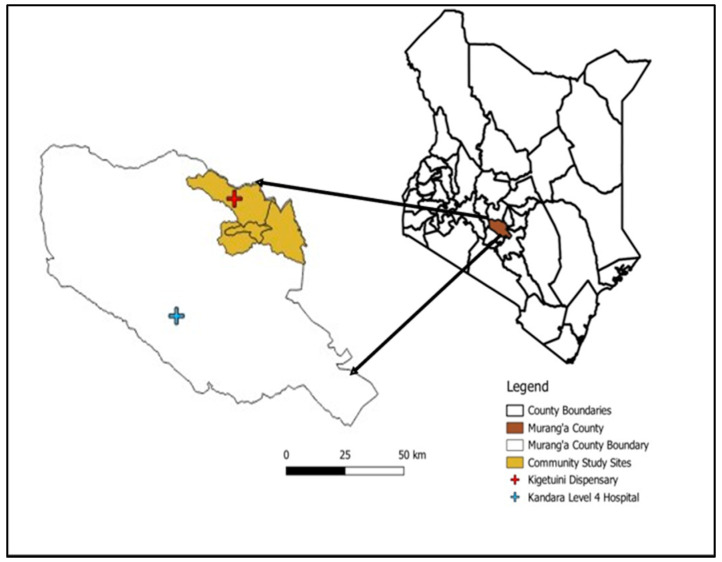
Map of Murang’a county, Kenya, showing locations where study participants were recruited.

Murang’a County, located in Kenya’s central highlands, is a high-altitude region (Figure 1) with elevations ranging from 914 to 3353 m above sea level, with most areas situated above 1200 m [19]. Farming is the main economic activity, with 80% of the population cultivating both food (maize, bananas) and cash crops (tea, coffee). Livestock rearing and mining are also practiced [19]. The hospital-based study was conducted at two healthcare facilities, Kandara Level III Sub-County Hospital and Kigetuini Level I dispensary, which were selected based on past reports of RVFV activity in Murang’a County. The cross-sectional human–animal study was conducted in Murang’a East sub-county, covering Gaturi, Mbiri, and Township wards. This site was purposively selected due to the higher RVFV IgG seropositivity of febrile patients detected in the hospital study.

### 2.3. Sampling Method and Sample Size

Febrile patients aged ≥10 years seeking care at the facilities for undifferentiated acute fever (≥37.5 °C at presentation or reported in the past four weeks), unexplained bleeding, or a severe illness of unknown etiology lasting ≥7 days despite treatment were eligible. We included 20% of malaria-positive cases to account for the overlap in risk factors between malaria and RVFV. Patients with specific infections (e.g., upper respiratory or urinary tract infections) and those with reported blood clotting disorders were excluded. A minimum sample size of 707 participants per healthcare facility was estimated based on an 8% RVFV prevalence in hotspots, 80% power, 2% precision, and a 95% confidence level [22].

A two-stage sampling approach was used in the community study. First, households were randomly selected, with the selection in each ward proportional to its population density. Within these households, individual livestock and humans served as the primary sampling units. Human participants were chosen at random, while livestock (cattle, sheep, and goats) were selected based on convenience. To identify study households, 350 random geographical coordinates in Murang’a East sub-county were generated using ArcGIS. This was based on a target sample size of 203, adjusted for a 30% decline rate and a 25% inaccessibility rate. Study teams used handheld GPS devices (Garmin eTrex^®^, Garmin, Olathe, KS, USA) and assigned geocodes to locate points. The nearest household within 100 m of each point was recruited. If no household was found or if the household head declined to participate, a backup point from a spare list was used. Assumed proportions were 5% for humans, 20.5% for cattle, and 5% each for sheep and goats [17]. A design effect (D) of 1.3 was applied to the livestock sample size using the formula D = 1 + (n − 1)ρ, where ρ is the intra-cluster correlation coefficient (ICC) and n is the average cluster size. For 2% precision and 95% confidence, we estimated a minimum sample size of 203 humans, 145 cattle, and 264 each of sheep and goats [23]. Both livestock (cattle, sheep, and goats) and non-livestock-owning households were eligible. For livestock-owning households, eligibility included owning at least one of the three livestock species (cattle, sheep, or goats), no history of RVFV vaccination, and a consenting human. All ages of livestock were eligible, with a maximum of two animals per species. In non-livestock-owning households, we sampled one consenting human, excluding children <1 year.

### 2.4. Participant Management

In the hospital-based study, participants who met the inclusion criteria were consented, given a physical examination, and had a questionnaire administered, and a blood sample was collected. Participants returned 4–6 weeks later for a convalescent blood draw and follow-up data collection.

In the community study, once a household was selected and consented, individuals within that household were listed then assigned numbers and a randomization software was used to select the individual to be included in the study. Only one individual per household was selected. The respondent was consented, and a questionnaire was administered to capture demographic and potential risk factors data at an individual and household level. For households with livestock, the owner was consented, and a maximum of two animals of either cattle, goats, or sheep were conveniently selected and animal-level data were collected using a questionnaire. Blood samples were collected from both humans and livestock.

### 2.5. Sampling Collection and Testing

**Figure 2:**
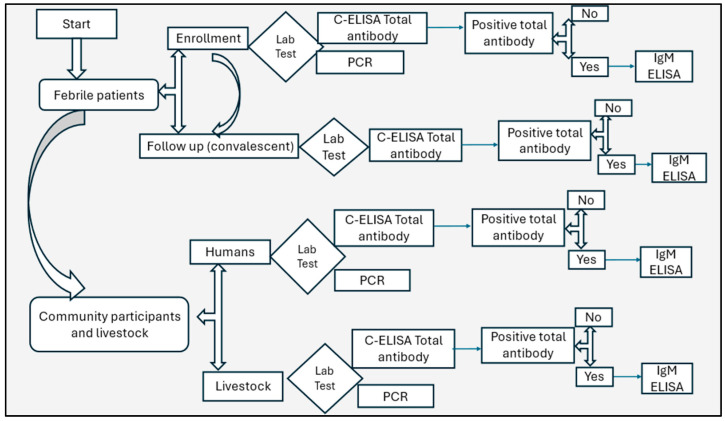
Flow chart showing RVFV serological and molecular diagnostics algorithm for the samples.

Four (4) mL of blood was collected from humans and six (6) mL from livestock using aseptic techniques into plain tubes. Samples were transported in a cold chain (2–8 °C) to the Kenya Medical Research Institute (KEMRI) laboratory in Nairobi, centrifuged, sera tested, aliquoted into barcoded cryovials, and stored at −20 °C. Samples were tested for RVFV total antibodies using a competitive enzyme-linked immunosorbent assay (C-ELISA), IgM antibodies using IgM capture ELISA (for total antibodies positive only), and RVFV RNA by a polymerase chain reaction (PCR), shown in Figure 2. Prior to testing, frozen samples were thawed and processed within 8–12 h to ensure the preservation of viral RNA.

### 2.6. Competitive Enzyme-Linked Immunosorbent Assay (C-ELISA)

The detection of IgG and IgM antibodies against the nucleoprotein (NP) was carried out using a multi-species RVFV competitive enzyme-linked immunosorbent commercial assay (C-ELISA) from IDvet^®^ (Grabels, France). Briefly, sera were diluted serially in duplicates and added to 96-well polystyrene plates that were precoated with a recombinant RVFV-NP. Test samples and controls were then added to the microwells. The anti-NP antibodies in the serum formed an antigen–antibody complex which masked the NP epitopes. Peroxidase-conjugated anti-NP antibodies were detected through a substrate-based absorbance (optical density) reading using a positive control as the standard. A nucleoprotein-peroxidase conjugate (Po) was added to the microwells to bind free NP epitopes and form an antigen–conjugate–peroxidase complex. After washing away excess conjugate, the substrate solution was added and finally, after incubating, the stop solution was added and absorbance was measured. The results were interpreted using the cut-off threshold specified by the manufacturer. All samples were run in duplicate, and the test was valid if the mean value of the positive control optical density (ODPC) was less than 30% of the negative control (ODNC), given as ODPC/ODNC < 0.3 and if the mean value of the negative control optical density (ODNC) was greater than 0.7, given as ODNC > 0.7. All runs met these criteria, and the indeterminate samples were considered negative. The test results were validated through duplicate assays. The IDvet kit reported diagnostic sensitivity and specificity rates of 98% and 100%, respectively [24].

### 2.7. Specific IgM ELISA Detection

All human and animal samples that tested antibody positive in the C-ELISA were re-tested for IgM antibodies. Human samples were tested using the Abbexa IgM capture ELISA (Grables, France), while livestock samples were tested using the ID Screen^®^ RVF IgM Capture Multi-species direct kit (IDvet Innovative Diagnostics, Grables, France), following the manufacturers’ instructions. Each sample was run in duplicate to investigate for IgM antibodies, which indicate recent infection. The test was valid if the mean optical density of the positive control (ODpc) was greater than 0.350, given as net ODpc > 0.350 and the ratio of the mean ODpc to the mean optical density for the negative control (ODnc) was greater than three, given as (net ODpc/|net ODnc|) > 3. Samples with suspect or positive (S/p) ≤ 40% were considered negative, samples with S/p ≥ 50% were considered positive, and anything <50% was negative.

### 2.8. PCR Assay

The molecular analysis for RVF viral RNA detection in the serum samples was con-ducted using a reverse transcription quantitative real-time PCR (RT-qPCR). RNA extraction was performed using the QIAamp Viral Mini Kit (Qiagen GmbH, Hilden, Germany) and the TANBead automated nucleic acid extraction system (TANBead Inc., Taoyuan, Tai-wan), according to the manufacturer’s instructions. We employed the One-Step RT–PCR Kit (Qiagen) for RNA amplification with primers targeting the L-segment of the virus: forward primer (RVFL-2912fwdGG) sequence was 5′-TGAAAATTCCTGAGA-CACATGG-3′, reverse primer (RVFL-2981revAC) was 5′-ACTTCCTTGCATCATCTGATG-3′, and probe (RVFL-probe-2950) was 5′-CAATGTAAGGGGCCTGTGTGGACTTGTG-3′ [25,26,27]. This primer–probe set amplified a 94-nucleotide fragment within a conserved domain of the L-segment. Optimization of the primers and probes included individual and multi-plex evaluations and limited detection studies with varied fluorescent dyes and quenchers. An RT-qPCR was performed on the RNA samples using the AgPath one-step reagent kit assay. The reactions used 5 µL (5–10 ng) of the RNA template in a 25 µL reaction volume using a final concentration of 0.3 µM for the primers and 0.1 µM for the probe in a PCR system (BioRad CFX 96 Maestro, Hercules, CA, USA). The reaction was carried out in a series of incubation steps as follows: 50 °C for 10 min, 95 °C for 2 min, 95 °C for 3 s, and 60 °C for 30 s for 40 cycles. Before testing the samples, we verified equipment equivalency through standard curve analysis and positive controls validation. Each sample was tested in triplicate wells to enhance accuracy. This assay uses a highly conserved domain located on the L-segment of the virus for RVFV detection (using 5′ Fam reporter dye and 3′ BHQ1 quencher dye). Samples were considered positive for RVFV RNA if the cycle threshold (Ct) value was ≤38, a cut-off determined through analytical sensitivity and clinical sensitivity assessment of the RT-qPCR assays using known positive samples.

### 2.9. RVF Virus Neutralizing Antibody Assay

A subset of 42 samples consisting of 26 positive, 10 negative, and 6 indeterminate by C-ELISA testing were subjected to the RVF virus neutralization test (VNT) as described previously [4,28,29,30]. Briefly, serial dilutions of heat-inactivated serum (1/50–1/1600 dilutions) were incubated with 3× TCID_100_ of RVF virus (strain h85/09, obtained from NCPV, UK Culture Collections) for 1 h at 37 °C before inoculating Vero cells in a 96-well plate. Development of the cytopathic effect (CPE) was scored microscopically and the Reed–Muench method was used to calculate the neutralizing antibody titer of by 50% (NT_50_) or 90% (NT_90_) of the cells [31].

### 2.10. Statistical Analysis

Continuous variables were summarized by mean, standard deviation (SD), median, and interquartile range (IQR), while categorical variables were summarized by proportions. RVFV seroprevalence and its 95% confidence interval were estimated using the “epi.prev” function in the EpiR package. Compared to other tools, epiR package provides accurate and reproducible seroprevalence estimates with exact confidence intervals and can adjust for complex sampling designs. The significance of factors associated with RVFV seropositivity was first determined using Fisher’s χ^2^ with a *p*-value < 0.05 considered statistically significant. Logistic regression was used for univariable analysis to identify variables of interest based on prior studies and biological plausibility (Supplementary Table S2). Variables with a *p*-value < 0.2 in univariable regression and other variables that did not fulfill this criterion, but where an association with RVFV IgG seemed likely due to biological reasons, were included in the multivariable analysis. The variables were then evaluated using a backward stepwise elimination and validated with the Hosmer–Lemeshow goodness-of-fit test. Explanatory variables were considered confounders if removing them from the model altered the coefficients of other significant variables by 30% or more. Serological status (positive or negative anti-RVFV IgG ELISA) was the dependent variable. Separate logistic regression models were developed for livestock and humans using the “glm” function in the lme4 R package. In addition, we assessed concordance between C-ELISA and VNT by Cohen’s Kappa, and prevalence and bias-adjusted kappa (PABAK) for 42 samples subjected to VNT. Cohen’s Kappa and PABAK values were interpreted based on the standard scale: “fair” agreement (Kappa = 0.21–0.40), “moderate” agreement (Kappa = 0.41–0.60), “substantial” agreement (Kappa = 0.61–0.80), and “almost perfect” agreement (Kappa > 0.80) [32]. All analyses were performed with R version 4.1.0 [33].

### 2.11. Ethical Approval

The study received ethical approval from the Kenya Medical Research Institute (KEMRI/SERU/CGHR/4169) and the Kenyatta National Hospital–University of Nairobi ethical review committee (P810/10/2022), and the National Commission for Science, Technology and Innovation [NACOSTI/P/23/24072]. Before enrollment, adult human participants provided written informed consent, while parental consent was obtained for children aged 1–9 years and assent for children aged 10–17 years. Livestock protocol was reviewed and approved by the KEMRI Institutional Animal Care and Use Committee (approval # KEMRI/ACUC/01.09.2021) and sampling consent obtained from the livestock owners.

## 3. Results

### 3.1. Characteristics of Human Participants

The study enrolled 1750 human participants (1468 febrile patients and 282 community members) with a median age of 37.1 (IQR: 25–49) years (Table 1). Of all the participants, 59.4% (n = 1040) attained primary level of education, 51.8% (n = 906) were female, 35.9% (n = 628) were engaged in farming, 10.5% (n = 184) were students, 9.1% (n = 160) were in formal employment, and 43.1% (n = 754) were involved in other occupation types (including business, transportation “boda boda”, and informal employment). Most (79.2%) participants reported owning livestock including cattle (73.0%), goats (60.4%), and sheep (8.6%). Almost all (94.0%, n = 1645) the participants reported contact with livestock, including feeding (77.8%), handling of raw meat during cooking (57.9%), and cleaning animal barns (57.9%) (Table 1).

### 3.2. Characteristics of Livestock

We sampled 706 animals in the community cross-sectional study, including 38.4% (n = 271) cattle, 49.0% (n = 346) goats, and 12.6% (n = 89) sheep from 255 households (Table 2). The majority (74.9%) (n = 529) of the animals were female while 67.2% (n = 182) were adults.

### 3.3. Environmental Risk Factor Analysis

Although 62.3% (n = 1091) of the participants reported not using any mosquito prevention, 83.5% (n = 1461) reported the presence of mosquitoes within their homesteads, as shown in Supplementary Table S1. In addition, 45.5% (n = 797) and 42.2% (n = 738) of the participants reported residing near quarries and wildlife, respectively. The association between independent risk factors and RVFV seropositivity is shown in Supplementary Table S1.

### 3.4. RVFV Prevalence in Humans

None of the sera from 1468 febrile human patients, and 282 asymptomatic community members, were positive for IgM or RVFV RNA by PCR. However, 34 of 1705 (2.0%, 95% CI: 1.38–2.78) were positive for anti-RVF virus IgG antibodies (Table 1). Butchers showed the highest seropositivity at 14.3%, (95% CI: 0.36–57.87), followed by farmers at 3.2% (95% CI: 1.96–4.88). As shown in Table 1, participants reporting animal contact including birthing, herding, milking, and slaughtering showed significantly higher RVFV seropositivity (*p* < 0.05). In addition, RVF seropositivity was significantly higher among participants residing near quarries, swamps, and wildlife. An important finding was that 4 of 1468 (0.27%, 95% CI: 0.07–0.69) febrile participants followed up 4–6 weeks later showed four-fold increase in IgG levels, suggesting recent RVFV exposure. Although animals had higher seropositivity rates, there were no significant associations between the types of livestock owned, contact with different livestock species, and human seropositivity (Supplementary Table S3). A univariate analysis was performed to assess whether animal seropositivity was associated with human RVFV seropositivity at the household level. The results indicated no significant association, with an odds ratio (OR) of 1.85 (95% CI: 0.04–19.8, *p* = 0.4777). We confirmed the ELISA-based seroprevalence results by performing a VNT on a subset of samples (N = 42) and found 80.5% concordance (29/36), including 73.0% concordance (19/26) among the positive and 100.0% among the negative (10/10) samples (Supplementary Figure S1). Cohen’s kappa (0.64) and PABAK (0.66) demonstrated substantial agreement between the ELISA and VNT assays.

### 3.5. RVFV Prevalence in Livestock

None of the sera from 706 livestock were positive for RVFV RNA by PCR. However, 4.4% (n = 31, 95% CI: 3.00–6.17) were positive for anti-RVF virus IgG antibodies, while at herd level, 24 of the 255 herds (9.4%, 95% CI: 6.12–13.68) had at least one positive animal (Table 2). Seropositivity was significantly different at 8.1% (95% CI: 5.16–12.03, *p* < 0.05) among cattle and 2.1% (95% CI: 0.95–3.89) among goats, while all sheep (n = 89) were seronegative. Higher seropositivity (5.5%, 95% CI: 1.24–11.11) was observed in females than males at 1.1% (95% CI: 0.14–4.02), and in adult (9.9%, 95% CI: 5.97–15.18) than young (4.5%, 95% CI: 5.16–12.03) animals.

### 3.6. Multivariable Analysis of Human Risk Factors of RVFV Seropositivity

The multivariable model fitted to the human data identified age, sex, animal contact, and proximity to quarries as significant predictors for RVFV seropositivity (Table 3). The model showed an increase in the odds of RVFV exposure with being male (aOR: 4.77, 95% CI: 2.08–12.4, *p*: <0.001) and residing near a quarry (aOR: 2.4, 95% CI: 1.08–5.72, *p*: 0.038). Respondents who engaged in milking were 2.69 times more likely to test positive for anti-RVFV IgG (aOR: 2.69, 95% CI: 1.23–6.36, *p*: 0.017) while those who consumed raw milk were 5.24 times more likely to be positive (aOR: 5.24, 95% CI: 1.13–17.9, *p*: 0.015).

### 3.7. Factors Associated with RVFV Seropositivity in Livestock

The multivariable model fitted to the animal data showed that small ruminants were 0.27 times less likely to be positive for RVFV IgG antibodies compared to cattle (aOR: 0.27, 95% CI: 0.12–0.65, *p*: 0.002) (Table 4). Breed, age, sex, and herd size did not show any associations with RVFV seropositivity.

## 4. Discussion

Although most highlands of East Africa lack the geo-ecological landmarks of RVF hotspots that are involved in historical RVF outbreaks in the East Africa region, recent studies have detected a growing number of small RVF disease cluster (<2 human and <5 livestock cases) in these highlands [16,34]. Therefore, this study was designed to investigate whether continuous cryptic RVF cycling occurred in a central highland of Kenya. The findings reported here document a seroprevalence of 4.4% in livestock and 2.0% in humans, levels comparable to those observed in regional non-RVF hotspots [15,35,36,37,38]. More importantly, we demonstrated evidence of recent RVFV exposure among 0.27% humans, illustrated by a four-fold increase in IgG antibodies in four febrile patients and 2.4% among young livestock (<12 months old), suggesting recurrent RVFV exposure in the area associated with cryptic circulation of the virus.

The significant human risk factors associated with RVFV exposure were the consumption of raw milk (aOR = 5.24), sex (aOR = 4.77), contact with animals (aOR = 2.69), and residing near a quarry (aOR = 2.4). These factors highlight the pathways through which RVFV primarily spreads, confirming that the long-held opinion that human-to-human transmission, if it occurs at all, has minimal public health significance. Despite limited data on milk’s infectious nature, recent studies have identified raw milk as a factor associated with RVFV exposure [10,15,39]. The consumption of raw milk suggests an alternative potential transmission route that may not require direct livestock contact. These findings are consistent with previously documented epidemiological patterns of RVFV [5,6,7,11,13,40,41].

Our results are consistent with some studies in Kenya showing similar seroprevalences during non-outbreak periods in other non-RVF hotspots, including the Lake Victoria region and in the western Kenya highlands [35]. The results contrast with studies from hyper-endemic regions with suitable habitats for RVFV vector survival. For instance, studies in Baringo, Isiolo, Tana River, and Garissa have found higher RVFV seroprevalence rates of 13 to 27%, which are likely due to a higher force of infection and virus circulation [5,6,7,13,14,40,42,43,44]. These differences could be due to variations in vector density ecologies that drive infection, low-risk practices, lower rates of infection, and sparse population demographics that minimize exposure risks [41,45]. The low seroprevalence observed may also be due to the possible clustering of cases in areas not yet investigated or activity due to a less virulent viral lineage [45].

We validated our serological findings from the C-ELISA assay by undertaking a confirmatory VNT, recording a concordance rate of 80.5%, similar to findings by previous studies [32,46,47]. A recent evaluation of the C-ELISA performance against a plaque reduction neutralization test estimated its diagnostic sensitivity at 0.854 [48], confirming the robustness of the C-ELISA test for RVFV antibody detection. The small variations in concordance rates arise from the different sensitivity and specificity of the assay used, as well as heterogeneity in the populations tested.

## 5. Conclusions

In conclusion, this study highlights sustained RVFV seroprevalence among humans and livestock in central highlands of Kenya and identifies key risk factors for exposure. The findings confirm the existence of a cryptic RVFV maintenance cycle in this ecosystem, which requires further studies to understand the mosquito vectors involved and the impact of climatic variability on the process. Taken in the context of other findings reporting increasing RVFV disease activities in the highlands, it represents a broader dispersal of the virus and a greater risk of more widespread RVFV epidemics in the future.

## Figures and Tables

**Table 1:** Demographic and RVFV exposure characteristics of human participants in Murang’a County, Kenya.

Variable	Levels	No. Sampled, N = 1750 ^1^ (%)	No. Negative = 1716 ^1^ (%)	No. Positive = 34 ^1^ (%)	Seropositivity Rate (%)	*p*-Value ^2^
**Sex**	Female	906 (51.8)	899 (52.4)	7 (20.6)	0.77	<0.001 *
	Male	844 (48.2)	817 (47.6)	27 (79.4)	**3.20**	
**Age**	Median (IQR)	37 (25, 49)	37 (25, 49)	48 (39, 59)	-	<0.001 *
**Education**	None	51 (2.9)	51 (3.0)	0 (0.0)	0.00	0.071
	Primary	1040 (59.4)	1015 (59.1)	25 (73.5)	2.40	
	Secondary	448 (25.6)	439 (25.6)	9 (26.5)	2.01	
	Tertiary	211 (12.1)	211 (12.3)	0 (0.0)	0.00	
**Occupation**	Butcher	7 (0.4)	6 (0.3)	1 (2.9)	**14.29**	0.006 *
	Farmer	628 (35.9)	608 (35.4)	20 (58.8)	3.18	
	Other	938 (53.6)	927 (54.0)	11 (32.4)	1.17	
	Formal	177 (10.1)	175 (10.2)	2 (5.9)	1.90	
**Keep livestock**	No	364 (20.8)	359 (20.9)	5 (14.7)	1.37	0.4
	Yes	1386 (79.2)	1357 (79.1)	29 (85.3)	2.09	
**Close animal contact**						
	No	105 (6.0)	105 (6.1)	0 (0.0)	0.00	0.3
	Yes	1645 (94.0)	1611 (93.9)	34 (100.0)	**2.07**	
**Types of close animal contact**						
**Birthing**	No	1596 (91.2)	1569 (91.4)	27 (79.4)	1.69	0.025 *
	Yes	154 (8.8)	147 (8.6)	7 (20.6)	**4.55**	
**Cleaning**	No	736 (42.1)	725 (42.2)	11 (32.4)	1.49	0.2
	Yes	1014 (57.9)	991 (57.8)	23 (67.6)	2.27	
**Feeding**	No	388 (22.2)	383 (22.3)	5 (14.7)	1.29	0.3
	Yes	1362 (77.8)	1333 (77.7)	29 (85.3)	2.13	
**Handling raw meat**						
	No	737 (42.1)	725 (42.2)	12 (35.3)	1.63	0.4
	Yes	1013 (57.9)	991 (57.8)	22 (64.7)	2.17	
**Herding**	No	1547 (88.4)	1522 (88.7)	25 (73.5)	1.62	0.012 *
	Yes	203 (11.6)	194 (11.3)	9 (26.5)	**4.43**	
**Milking**	No	1081 (61.8)	1072 (62.5)	9 (26.5)	0.83	<0.001
	Yes	669 (38.2)	644 (37.5)	25 (73.5)	3.74	
**Slaughtering**	No	1620 (92.6)	1594 (92.9)	26 (76.5)	1.60	0.003 *
	Yes	130 (7.4)	122 (7.1)	8 (23.5)	**6.15**	
**Spraying**	No	1455 (83.1)	1437 (83.7)	18 (52.9)	1.24	<0.001 *
	Yes	295 (16.9)	279 (16.3)	16 (47.1)	**5.42**	
**Treating**	No	1730 (98.9)	1697 (98.9)	33 (97.1)	1.91	0.3
	Yes	20 (1.1)	19 (1.1)	1 (2.9)	**5.00**	

^1^ n (%); ^2^ Pearson’s chi-squared test; Fisher’s exact test; * statistically significant; bold text indicates the category with the highest seropositivity.

**Table 2:** Demographic and RVFV exposure characteristics of livestock enrolled in the community-based serosurvey in Murang’a County, Kenya.

Variable	No. Sampled = N = 706 ^1^ (%)	No. Negative = 675 ^1^ (%)	No. Positive = 31 ^1^ (%)	Seropositivity Rate (%)	*p*-Value ^2^
**Ward**					0.2
Gaturi	298 (42.2)	286 (42.4)	12 (38.7)	4.03	
Mbiri	272 (38.5)	256 (37.9)	16 (51.6)	**5.88**	
Township	136 (19.3)	133 (19.7)	3 (9.7)	2.21	
**Species**					<0.001 *
Cattle	271 (38.4)	249 (36.9)	22 (71.0)	**8.12**	
Shoats	435 (61.6)	426 (63.1)	9 (29.0)	2.07	
**Sex**					0.014 *
Female	529 (74.9)	500 (74.1)	29 (93.5)	**5.48**	
Male	177 (25.1)	175 (25.9)	2 (6.5)	1.13	
**Breed**					0.3
Cross	150 (21.2)	147 (21.8)	3 (9.7)	2.00	
Exotic	381 (54.0)	362 (53.6)	19 (61.3)	4.99	
Local	175 (24.8)	166 (24.6)	9 (29.0)	5.14	
**Cattle age**					0.13
Adult	182 (67.2)	164 (65.9)	18 (81.8)	**9.89**	
Young (<12 months)	89 (32.8)	85 (34.1)	4 (18.2	4.49	
**Shoat’s age**					0.5
Adult	316 (72.6)	308 (72.3)	8 (88.9)	**2.53**	
Young (<12 months)	119 (27.4)	118 (27.7)	1 (11.1)	0.84	
**Herd size**					0.2
>5	356 (50.4)	337 (49.9)	19 (61.3)	**5.34**	
1—5	350 (49.6)	338 (50.1)	12 (38.7)	3.43	

^1^ n (%); ^2^ Pearson’s chi-squared test; Fisher’s exact test; * statistically significant; bold text indicates the category with the highest seropositivity.

**Table 3:** Regression analysis for factors associated with RVFV seropositivity among humans in Murang’a County, Kenya.

Multivariable Model				
Characteristic	Variable	aOR	95% CI ^1^	*p*-Value
Age	-	1.05	1.03, 1.07	<0.001 *
Gender	Female (Ref)	—	—	
	Male	4.77	2.08, 12.4	<0.001 *
Milking animals	No (Ref)	—	—	
	Yes	2.69	1.23, 6.36	0.017 *
Drinking raw milk	No (Ref)	—	—	
	Yes	5.24	1.13, 17.9	0.015 *
Abortions in herds	No (Ref)	—	—	
	Yes	2.26	0.88, 5.38	0.074
Keeping cattle	No (Ref)	—	—	
	Yes	3.12	0.89, 19.8	0.13
Presence of a quarry	No (Ref)	—	—	
	Yes	2.4	1.08, 5.72	0.038 *
Mosquito net use	No (Ref)	—	—	
	Yes	0.51	0.20, 1.16	0.13

aOR = adjusted odds ratio, ^1^ CI = confidence interval; * statistically significant.

**Table 4:** Regression analysis for factors associated with RVFV seropositivity among livestock (n = 706) in Murang’a County, Kenya.

Multivariable Model				
Characteristic	Variable	aOR	95% CI ^1^	*p*-Value
Livestock species	Cattle (Ref)	—	—	
	Small ruminants	0.27	0.12, 0.60	0.002 *
Breed distribution	Cross (Ref)	—	—	
	Exotic	1.79	0.57, 7.85	0.4
	Local	2.28	0.64, 10.6	0.2
Animal age	Adult > 12 months (Ref)	—	—	
	Young < 12 months	0.5	0.16, 1.24	0.2
Sex	Female (Ref)	—	—	
	Male	0.29	0.05, 1.04	0.1
Livestock herd size	>5 animals (Ref)	—	—	
	1–5 animals	0.6	0.27, 1.26	0.2

aOR = adjusted odds ratio, ^1^ CI = confidence interval; * statistically significant.

## Data Availability

The raw data used to support the findings of this study can be provided by the authors on request.

## Supplementary Materials

The following supporting information can be downloaded at: https://www.mdpi.com/article/10.3390/v16121927/s1, Table S1. Environmental risk factors for RVFV seropositivity; Table S2. Livestock risk factors for RVFV seropositivity; Table S3. Predictor variables collected and their measurements; Figure S1. Correlation plots of ELISA OD values/SN ratios against neutralization assay titers NT_50_ and NT_90_ for the samples.
