# Supplementary material for: Serological Evidence of Cryptic Rift Valley Fever Virus Transmission Among Humans and Livestock in Central Highlands of Kenya
Source: Viruses. 2024 Dec 17;16(12):1927. doi: 10.3390/v16121927 (PMC11680181; doi:10.3390/v16121927)
Supplement: Supplementary file 1 [file viruses-16-01927-s001.zip › viruses-3273207-supplementary.pdf]

Table S1. Environmental risk factors for RVFV seropositivity.

Variable	Total sampled, N = 1,750[1] (%)	No. Negative = 1,716[1] (%)	No. Positive = 34[1] (%)	Seropositivity rate (%)	p-Value
Presence of mosquitoes					0.5
No	289 (16.5)	285 (16.6)	4 (11.8)	1.38	
Yes	1,461 (83.5)	1,431 (83.4)	30 (88.2)	**2.05**	
Use of treated mosquito net					0.8
No	1,589 (90.8)	1,557 (90.7)	32 (94.1)	2.01	
Yes	161 (9.2)	159 (9.3)	2 (5.9)	1.24	
Use of repellant					0.3
No	1,654 (94.5)	1,620 (94.4)	34 (100.0)	2.06	
Yes	96 (5.5)	96 (5.6)	0 (0.0)	0.00	
No mosquito prevention					0.3
No	659 (37.7)	649 (37.8)	10 (29.4)	1.52	
Yes	1,091 (62.3)	1,067 (62.2)	24 (70.6)	**2.20**	
Presence of quarry					<0.001*
No	953 (54.5)	944 (55.0)	9 (26.5)	0.94	
Yes	797 (45.5)	772 (45.0)	25 (73.5)	**3.14**	
Presence of swamp					0.006*
No	975 (55.7)	964 (56.2)	11 (32.4)	1.13	
Yes	775 (44.3)	752 (43.8)	23 (67.6)	**2.97**	
Flooding in past 2 months					0.7
No	1,667 (95.3)	1,635 (95.3)	32 (94.1)	1.92	
Yes	83 (4.7)	81 (4.7)	2 (5.9)	**2.41**	
Wildlife in proximity					0.002*
No	1,012 (57.8)	1,001 (58.3)	11 (32.4)	1.09	
Yes	738 (42.2)	715 (41.7)	23 (67.6)	**3.12**	
Presence of irrigation scheme					>0.9
No	1,595 (91.1)	1,564 (91.1)	31 (91.2)	1.94	
Yes	155 (8.9)	152 (8.9)	3 (8.8)	1.94	
Travel outside residential area					0.3
No	1,543 (88.2)	1,515 (88.3)	28 (82.4)	1.81	
Yes	207 (11.8)	201 (11.7)	6 (17.6)	**2.90**	
Use Rainwater in household					0.001*
No	1,080 (61.7)	1,068 (62.2)	12 (35.3)	1.11	
Yes	670 (38.3)	648 (37.8)	22 (64.7)	**3.28**	

[1] n (%); [2] Pearson's Chi-squared test; Fisher's exact test, * Statistically significant.

Table S2. Livestock risk factors for RVFV seropositivity.

Characteristic	Total sampled N = 1,750[1] (%)	No. Negative = 1,716[1] (%)	No. Positive = 34[1] (%)	Seropositivity rate (%)	p-value[2]
**Livestock contact by species**					0.5
Cattle, sheep and goats	1,073 (61.3)	1,047 (61.0)	26 (76.5)	2.42	
Cattle only	378 (21.6)	372 (21.7)	6 (17.6)	1.59	
Goats only	176 (10.1)	174 (10.1)	2 (5.9)	1.14	
Sheep and goats	10 (0.6)	10 (0.6)	0 (0.0)	0.00	
Sheep only	8 (0.5)	8 (0.5)	0 (0.0)	0.00	
None	105 (6.0)	105 (6.1)	0 (0.0)	0.00	
**Livestock species kept**					0.6
Cattle and goats	572 (32.7)	557 (32.5)	15 (44.1)	2.62	
Cattle only	423 (24.2)	417 (24.3)	6 (17.6)	1.42	
Cattle, sheep and goats	56 (3.2)	55 (3.2)	1 (2.9)	1.79	
Goats only	277 (15.8)	271 (15.8)	6 (17.6)	2.17	
Cattle and sheep	19 (1.1)	18 (1.0)	1 (2.9)	5.26	
Sheep only	13 (0.7)	13 (0.8)	0 (0.0)	0.00	
None	364 (20.8)	359 (20.9)	5 (14.7)	1.37	
**Abortion in herds**					0.002*
No	1,626 (92.9)	1,600 (93.2)	26 (76.5)	1.60	
Yes	124 (7.1)	116 (6.8)	8 (23.5)	**6.45**	
**Drink raw milk**					0.017*
No	1,721 (98.3)	1,690 (98.5)	31 (91.2)	1.80	
Yes	29 (1.7)	26 (1.5)	3 (8.8)	**10.34**	
**Eat wild meat**					0.13
No	1,650 (94.3)	1,620 (94.4)	30 (88.2)	1.82	
Yes	100 (5.7)	96 (5.6)	4 (11.8)	**4.00**	

[1] n (%); [2] Pearson's Chi-squared test; Fisher's exact test, * Statistically significant.

Table S3. Predictor variables collected and their measurements.

Variable (Type)	Measurement
Healthcare facility (nominal)	Assessed in two levels: Kandara & Kigetuini
Study wards (nominal)	Assessed in three levels: Mbiri, Gaturi & Township
Age (continuous)	Captured in years
Sex (nominal)	Entered as male or female
Marital status (nominal)	Assessed in five levels: single, separated, divorced, NA(Child), married
Occupation type (nominal)	Assessed in five levels: Farmer, Butcher, formal, unskilled, other
Education levels (nominal)	Assessed in two levels: Formal and Informal
Travel out of residential area	Assessed in two levels: Yes or No
Type of close contact	
Herding	Assessed in two levels: Yes or No
Milking	Assessed in two levels: Yes or No
Birthing	Assessed in two levels: Yes or No
Slaughter	Assessed in two levels: Yes or No
Cleaning animal barns	Assessed in two levels: Yes or No
Feeding animals	Assessed in two levels: Yes or No
Sharing same room with animals	Assessed in two levels: Yes or No
Spraying animals	Assessed in two levels: Yes or No
Treating animals	Assessed in two levels: Yes or No
Slaughter of dead animals	Assessed in two levels: Yes or No
Contact with animals	Assessed in two levels: Yes or No
Animal species with close contact (nominal)	Assessed in three levels: cattle, sheep and goats
Drink raw milk	Assessed in two levels: Yes or No
Eat raw meat	Assessed in two levels: Yes or No
Eat wild meat	Assessed in two levels: Yes or No
Presence of mosquitoes in area	Assessed in two levels: Yes or No
Types of mosquito prevention	Assessed in five levels: mosquito net, repellant, clearing bushes, other, none
Mosquito prevention	Assessed in two levels: Yes or No
Own livestock	Assessed in two levels: Yes or No
Type of livestock owned	Assessed in three levels: cattle, sheep, goats
Proximity to wildlife, swamps or irrigation schemes	Assessed in two levels: Yes or No
Sources of household water	Assessed in four levels: piped, well, river, rainwater and bottled water
RVFV seropositivity	0=negative, 1=positive
Livestock abortion reports	Assessed in two levels: Yes or No
Livestock death reports	Assessed in two levels: Yes or No
Presence of flooding	Assessed in two levels: Yes or No
Presence of heavy rainfall in the area	Assessed in two levels: Yes or No
Livestock	

Study wards (nominal)	Assessed in three levels: Mbiri, Gaturi & Township
Age (nominal)	Assessed in three levels: Adults, juveniles, infants
Sex (nominal)	Entered as male or female
Breed	Assessed in three levels: exotic, indigenous, cross
Species	Assessed in three levels: Cattle, sheep and goats
RVFV vaccination status	Assessed in two levels: Yes, No
Herd size	Assessed in two levels: 1-5 animals, > 5 animals
Yearling suckling status	Assessed in two levels: Yes, No
Presence of livetsock abortions	Assessed in two levels: Yes, No
Ill health in livetsock	Assessed in two levels: Yes, No
Presence of livestock still deaths	Assessed in two levels: Yes, No
Livetsock production system type	Assessed in three levels: intensive, semi-intensive, extensive

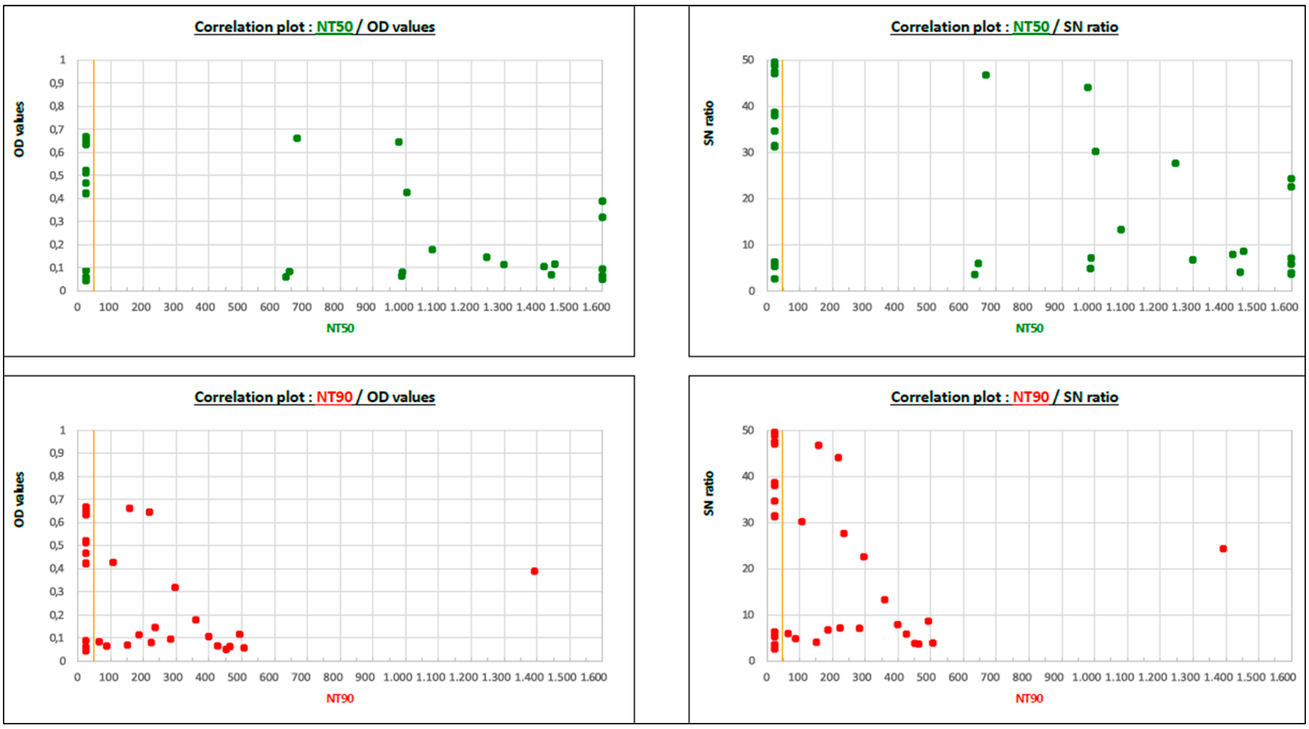

Figure S1. Correlation plots of ELISA OD values/SN ratios against neutralization assay titres NT_{50} and NT_{90} for the samples.
